# Associations between absolute and relative handgrip strength with fitness and fatness

**DOI:** 10.1055/a-2537-7537

**Published:** 2025-04-22

**Authors:** David Abdelnour, Mark Grove II, Keegan Pulford-Thorpe, Keaton Windhurst, Charlee LeCrone, Edward Kerr III, Tamara Hew-Butler

**Affiliations:** 1 School of Medicine, Wayne State University, Detroit, United States; 2 School of Business, Wayne State University, Detroit, United States; 3 Kinesiology, Health and Sport Studies, Wayne State University, Detroit, United States

**Keywords:** body composition, muscle strength, fitness assessment

## Abstract

The main purpose of this study was to assess relationships between absolute and relative handgrip strength (HGS) versus other markers of health (body composition) and physical fitness (VO _2_ max, vertical jump) in 220 (112 male) healthy young adults (25±10 years). HGS was measured using a hand dynamometer. Absolute HGS represented the highest grip strength measurement (kg) of the right and left hand combined, while relative HGS represented the absolute HGS divided by body weight (kg/kg). Body composition (lean and fat mass) was measured using dual energy x-ray absorptiometry. VO _2_ max was measured using a treadmill peak speed protocol (ml/kg/min), while vertical jump was assessed using a countermovement jump (cm). Absolute HGS (mean=86±22 kg) was positively related with lean mass (r=0.82, p<0.001) and vertical jump (r=0.63, p<0.001), while relative HGS (mean=1.2±0.2 kg/kg) was negatively related with body fat (r=–0.69, p<0.001), but positively correlated with VO _2_ max (r=0.47, p<0.001), and vertical jump (r=0.45, p<0.001). Linear models suggest that lean mass, body fat, and vertical jump predicted 69% of variance for absolute HGS (adjusted R ^2^ =0.71, p<0.001), while lean mass and body fat predicted 49% of variance for relative HGS (adjusted R ^2^ =0.49, p<0.001). Lower relative HGS scores (<1.0 kg/kg) were associated with higher body fat levels and may represent a quick, simple, marker of health.

## Introduction

Handgrip strength (HGS) is a quick, simple, and portable measure of generalized muscle strength and physical functioning, particularly in elderly populations 1 2 3 . However, the measurement of maximum isometric handgrip force is often applied in a vague and inconsistent manner across populations. Epidemiological studies, largely conducted in older populations, confirm that low absolute HGS is a surrogate marker for muscle weakness, which are then strongly associated with cardiovascular disease 2 , dementia 4 , cancer 5 , disability 3 , and all-cause mortality 1 2 . More clinically relevant HGS thresholds remain under-developed, however, due to a lack of both clarity and standardization of measurement. For example, maximum HGS thresholds of 42 kg for males and 25 kg for females were estimated to reduce all-cause mortality in a prospective study performed on ~64-year-old adults from 28 countries 6 . Whether or not these mortality thresholds represent a single HGS or both HGSs combined remains unclear.

Thus, the difficulty interpreting HGS – and the practical application of these values – is largely due to the high variability in reporting. While the reporting of absolute HGS values is common, the reporting of single hand (right or left) 7 8 versus double hand (right plus left hand) 4 5 or using the average score 4 5 versus the highest score 9 alter the practical utility of HGS across studies. Furthermore, HGS has also been reported in Newtons 3 10 , quartiles 4 5 11 , quintiles 3 , and/or normalized by dividing absolute HGS by body mass index (BMI) 11 12 and body weight 13 14 . Thus, although evidence supports that low HGS is associated with death, disease, and disability the clinical utility of providing a unified, practical, metric to assess health in real time is lacking.

In contrast to the robust amount of data obtained from older populations, the utility of HGS to predict health and disease in younger populations remains under-investigated. In (young) elite athletic populations, absolute HGS is related with upper and lower body strength, impulsive (jumping) ability, body mass, lean muscle mass, age, and training experience 15 . Of greater clinical utility, the normalization of HGS, by dividing absolute HGS by body weight, has produced cut-off thresholds that predict both diabetes risk 13 and metabolic syndrome (MetS) 14 in middle-aged adults. Thus, relative HGS (rather than absolute HGS) may be a more practical and universally applied metric to assess fitness, health and/or disease risk in non-elderly populations.

Therefore, the main purpose of this study is to assess relationships between both absolute and relative HGS versus markers of metabolic health (blood pressure, resting heart rate, fasted blood glucose, and visceral adipose tissue), physical health (body composition), and performance (VO _2_ max, vertical jump) in a convenience sample of young, healthy, adults. A secondary purpose is to establish a predictive model, for both absolute and relative HGS, utilizing these select markers of health (physical and metabolic) and (fitness) performance. A tertiary purpose is to evaluate mean thresholds for HGS that would translate across average health and fitness metrics in young adults.

## Methods

### Participants

This observational, cross-sectional study (IRB#073919M1E) recruited a convenience sample of both male and female participants from Detroit and the greater metropolitan area. The inclusion criteria represented any able-bodied individual (i. e., who can run on a treadmill until volitional exhaustion) between the ages of 18–100 years old. Any participant with medical conditions that may be exacerbated by running on a treadmill to volitional exhaustion (VO ^2^ max test), were asked to obtain clearance from their medical practitioner before participating in this study. The only exclusion criteria was any menstruant female who was pregnant (or thought they might be pregnant), due to radiation exposure concerns from the dual energy x-ray absorptiometry – or DXA – scan, which may be harmful to a developing fetus. This study served to provide a “snapshot” of baseline metrics, to launch ongoing longitudinal investigations (and interventions) for local community members.

All participants were asked to arrive at the laboratory in exercise attire after a 4-hour fast (i. e., no food or drink, other than water when thirsty) and sign written informed consent prior to participation. All females of child-bearing age were asked to complete a pregnancy attestation form, confirming that they were not pregnant (or think they were pregnant) prior to participation.

We assessed metabolic health through measurement of four parameters: resting blood pressure (BP), resting heart rate (RHR), fasted blood glucose (BG), and visceral adipose tissue (VAT; obtained from the DXA scan). We assessed physical health via whole body composition analyses (measuring three compartments: lean, fat, and bone mass). We assessed physical fitness (i. e., performance) using three different tests that measured: cardiorespiratory fitness (VO _2_ max), muscular strength (handgrip), and muscular power (vertical jump height). The exact procedures are detailed below, as represented sequentially, in our ~60-minute study protocol.

### Protocol

After written informed consent was obtained, each participant sat quietly for 5-minutes to achieve a resting, steady-state condition prior to blood pressure (BP) measurement. With both feet flat on the ground, back upright, with both forearms placed supine on the table, resting blood pressure was measured once on the right arm using an automated BP cuff (OMRON 3, Kyoto, Japan). Systolic blood pressure (SBP), diastolic blood pressure (DBP), and resting heart rate (RHR) were recorded.

Next, still in a seated position, a fingerstick fasting blood glucose (BG) was measured using a portable analyzer (GE100 Blood Glucose Monitoring Kit, Ontario, CA) and aseptic technique.

Height and weight were measured using a stadiometer and electronic scale (SECA 763, Hamburg, Germany), with participants wearing only compression shorts and a sports bra (females), without shoes. Body composition was assessed using a whole body DXA scan (Horizon A, APEX System software version 5.6.0.5, TBAR2019 calibration; Hologic, Marlborough, MA). All DXA scans were performed and analyzed by a single trained operator, according to the manufacturer’s specifications 16 .

After body composition measurement, handgrip strength (HGS) was measured using a digital hand dynamometer (Handexer, South El Monte, CA). In a standing position, both elbows were simultaneously flexed to a 90º angle with the wrists held in a neutral position. Maximal HGS was measured, with verbal encouragement, per hand (3-second max contractions, with a 30-second rest between trials, alternating the dynamometer between hands). Both hands were tested three times, with the sum of the highest value (kg), for both the right and left hands, representing the absolute HGS score 17 . Relative HGS (absolute HGS in kg divided by body weight in kg) was also calculated to normalize strength per body mass, as calculated previously 13 14 .

A standing vertical jump test was then performed, using a Vertec jump trainer (Vertec Gen2, Cranston, RI). The highest of three countermovement jump attempts was recorded. We chose to report vertical jump in inches, to compare with other normative values (measured in inches) more easily but also converted to peak power output using the following equation: peak power (watts)=60.7 x (jump height [cm])+45.3 x (body mass [kg]) – 2055.

Lastly, aerobic fitness (VO _2_ max) was assessed on a motorized treadmill (Lode Valiant 2 Sport, Groningen, Netherlands). Oxygen uptake was measured continuously using a metabolic cart (Parvo Medics TrueOne 2400, Sandy, UT) with maximal oxygen consumption defined as the highest value obtained before volitional exhaustion. The exercise protocol utilized was a peak treadmill speed running test, whereas all participants started at a comfortable walking or running speed. After 1 min, the treadmill speed increased 0.5 mph every minute until participants could no longer keep pace with the treadmill (volitional exhaustion) 18 .

### Statistical analysis

Unpaired t-tests were used to confirm expected sex differences in health and performance metrics. Simple regression analyses (Pearson’s r) were utilized to assess relationships between our two main outcome measures, absolute HGS and relative HGS, versus indicators of metabolic and physical health as well as physical performance. Prediction equations, for both absolute and relative HGS (dependent variables), were generated using stepwise linear regression models. The independent variables demonstrating the strongest statistical significance with the two dependent variables ( Table 3 ) were sequentially added into the general linear model. The final prediction models, for both absolute and relative HGS, included only those independent variables that retained statistical significance (p<0.05) as predictors within the linear model. The adjusted R ^2^ for each linear model reflected the variance that the combined independent variables contribute to each dependent variable (i. e., absolute and relative HGS).

**Table:** **Table 3** Correlation coefficients (r) for analyses comparing both absolute HGS and relative HGS versus demographic (age, BMI), body composition (LM, BF, Z-score), metabolic (VAT, SBP, DBP, and BG), and physical performance (VO2, VJ) variables, for combined data (N=220). BMI=body mass index; LM=lean mass; BF=body fat; VAT=visceral adipose tissue; SBP=systolic blood pressure; DBP=diastolic blood pressure; RHR=resting heart rate; BG=blood glucose; VO2=VO2 max; VJ=vertical jump.

Handgrip Strength	Age *(years)*	BMI *(kgm* ^*2*^ *)*	LM *(kg)*	BF *(%)*	Z- score	VAT *(cm* ^*2*^ *)*	SBP *(mmHg)*	DBP ( *mmHg)*	RHR *(bpm)*	BG *(mg/dL)*	VO _2_ *(ml/kg/min)*	VJ *(cm)*
**Absolute** ***(kg)***	–0.05	0.34***	0.82***	–0.53***	0.22*	0.20***	0.49***	0.02	–0.09	0.08	0.21**	0.63***
**Relative** ***(kg/kg)***	–0.09	–0.37***	0.20**	–0.69***	0.01	–0.35	0.22**	–0.08	–0.13	0.04	0.47***	0.45***

^*^ p<0.05; **p<0.01; ***p<0.001

Effect sizes to determine the magnitude of effects 19 were expressed using Cohen’s *d* with small (0.2), medium (0.5), and large (0.8) magnitude of effects calculated for both the unpaired t-tests (male mean – female mean/pooled SD) and regression correlations (using the r-value/ correlation coefficient) 20 . A power analysis, conducted on a similar cross-sectional study assessing relationships between HGS and VO _2_ max on a convenience sample of male and female elderly outpatients, calculated a sample size of 160 participants necessary to achieve 95% power, α=0.05, β=0.05, with an anticipated effect size=0.15 (G*Power 3.1.9.2.; Heinrich-Heine-Universität, Düsseldorf, Germany) 8 . All data reported as mean±standard deviation (SD), with statistical significance set *a priori* at p<0.05. Both Cohen’s *d* and statistical significance were reported, offering two distinct perspectives on the translational (practical) interpretation of “significance.”

## Results

A total of 220 (112 male, 51% and 108 female, 49%) participants completed the full testing protocol. In our reporting of means (±SD), we have separated our cohort into males and females to confirm the expected sex-specific differences well-documented in body composition and performance metrics verified and reported elsewhere 17 . The average demographic (age, height, weight, body mass index/BMI), body composition (lean, fat, and bone mass), and metabolic health (VAT, SBP, DBP, RHR, BG) metrics for males, females, and the total cohort combined are summarized in Table 1 . As expected, large effects sizes (>0.8 for Cohen’s *d* ) were noted between male and female participants with respect to sex-related differences in lean mass, body fat percentage, and systolic blood pressure.

**Table:** **Table 1** Demographic (age, height, weight, body mass index/BMI), body composition (lean, fat, and bone mass) and metabolic (VAT, resting heart rate, blood pressure, and blood glucose) data for all (total; N=220), male (n=112), and female (n=108) participants. Data expressed as mean±SD (min-max). The magnitude of difference between male and female participants are demonstrated using both the p-values (from unpaired t-tests) and effect size (from Cohen’s *d* ). BMI=body mass index; BMD=bone mineral density; VAT=visceral adipose tissue; BP=blood pressure.

Variable	Total	Male	Female	p-value	Cohen’s *d*
**Age** ***(years)***	25±10 *(18–64)*	25±9	25±11	0.59	0.06
**Height** ***(m)***	1.71±0.10 *(1.5–2.0)*	1.77±0.08	1.65±0.06	<0.001	1.2
**Weight** ***(kgs)***	72.9±15.1 *(43–129)*	80.0±14.7	65.6±11.6	<0.001	0.95
**BMI** ***(kg/m*** ^***2***^ ***)***	24.7±4.1 *(17–41)*	25.3±4.1	24 .0±3.9	0.01	0.35
**Total lean mass** ***(kg)***	53.3±12.2 *(51–85)*	61.6±10.1	44.8±7.3	<0.001	1.38
**Total body fat** ***(%)***	24.4±7.2 *(12–47)*	19.9±5.2	28.9±6.0	<0.001	1.25
**BMD Z-score** ***(no units)***	–0.1±0.9 *(–2.5–2.4)*	–0.1±0.8	–0.1±0.9	0.75	0.05
**VAT** ***(cm*** ^***2***^ ***)***	52.5±23.5 *(18–195)*	60.6±19.4	44.2±24.5	<0.001	0.70
**Systolic BP** ***(mmHg)***	122±15 *(87–168)*	129±14	115±9	<0.001	0.90
**Diastolic BP** ***(mmHg)***	75±9 *(49–106)*	76±14	76±14	0.88	0.00
**Resting heart rate** ***(bpm)***	75±14 *(39–118)*	73±14	76±13	0.08	0.21
**Blood glucose** ***(mg/dL)***	86±14 *(46–136)*	86±15	85±13	0.80	0.07

Table 2 shows the mean performance metrics of all (total), male, and female participants. As expected, large effect sizes (>0.8 for Cohen’s *d* ) between male and female participants were seen in absolute and relative HGS, vertical jump, and peak power.

**Table:** **Table 2** Physical performance metrics data for all (Total), male, and female participants. Data expressed as mean±SD (min-max). The magnitude of difference between male and female participants are demonstrated using both the p-values (from unpaired t-tests) and effect size (from Cohen’s *d* ).

Variable	Total	Male	Female	p-value	Cohen’s *d*
**Absolute handgrip** ***(kg)***	86±22 *(31–144)*	102±17	70±14	<0.001	1.45
**Vertical jump** ***(cm)***	51±15 *(15–104)*	58±13	43±10	<0.001	1.00
**Peak power** ***(Watts)***	4348±1275 *(1304–8656)*	5178±1094	3489±789	<0.001	1.32
** VO _2_ max ** ***(ml/kg/min)***	43±10 *(21–83)*	47±11	40±11	<0.001	0.70
**Relative handgrip** ***(kg/kg)***	1.2±0.2 *(0.6–1.8)*	1.3±0.2	1.1±0.2	<0.001	1.00

Table 3 details linear relationships between our two main outcome measures, absolute and relative HGS, versus demographic (age, BMI), body composition (lean mass, % body fat, and BMD Z-score), metabolic health (VAT, SPB, DBP, and BG), and performance (VO2 max, vertical jump) metrics.

**Fig. 1:**
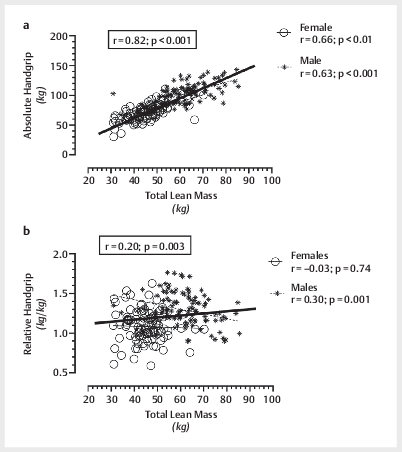
Relationships between absolute (total handgrip or right plus left) handgrip score versus whole body lean mass ( **a** ) and relative (total handgrip score/body weight) handgrip score versus whole body lean mass ( **b** ) for males, females, and the combined cohort.

**Fig. 2:**
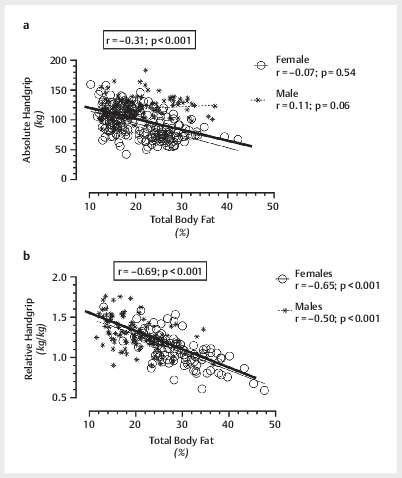
Relationships between absolute (total handgrip or right plus left) handgrip score versus total body fat percentage ( **a** ) and relative (total handgrip score/body weight) handgrip score versus total body fat percentage ( **b** ) for males, females, and the combined cohort.

Fig. 1 demonstrates correlations between absolute HGS ( Fig. 1a ) and relative HGS ( Fig. 1b ) versus total lean mass, for the total cohort and separated by sex. Fig. 2 demonstrates correlations between absolute HGS ( Fig. 2a ) and relative HGS ( Fig. 2b ) versus total body fat percentage, for the total cohort and separated by sex. A large effect size was noted when male and female participants were combined between absolute HGS versus total lean mass ( Fig. 1a ), while a medium to large effect size was noted between relative HGS versus body fat% ( Fig. 2b ).

Stepwise linear regression models identified three significant predictors for absolute HGS: lean mass, vertical jump height, and body fat (multiple R=0.84; adjusted R ^2^ =0.71; F=176.99; p<0.001). From this linear model, the prediction equation for absolute HGS is:

Absolute HGS (kg)=18.62–0.40 (body fat in %)+1.22 (lean mass in kg)+0.24 (vertical jump height in cm). Thus, more practically speaking, every 0.4% decrease in body fat or 1.2 kg increase in lean mass, or 0.24 cm increase in vertical jump height would result in a 1 kg increase in absolute HGS.

Stepwise linear regression identified two significant predictors for relative HGS: body fat and lean mass (multiple R=0.69; adjusted R ^2^ =0.49; F=104.6; p<0.001). From this linear model, the prediction equation for relative HGS is:

Relative HGS (kg/kg)=1.90–0.2 (body fat in %) – 0.002 (lean mass in kg). Thus, more practically speaking, every 0.2% decrease in body fat or 0.002 decrease in lean mass would result in an increase in relative HGS.

## Discussion

The main conclusions derived from this study suggests that absolute HGS is strongly and positively related to lean mass, while relative HGS is strongly and negatively related to body fat percentage, in this cohort of young (~25 years old), metabolically healthy ( Table 1 ), and physically fit ( Table 2 ) individuals. The robust relationship seen in the current study between absolute HGS versus total lean mass ( Fig. 1a ) complements previous studies that confirm that low absolute HGS is associated with sarcopenia 21 , muscle weakness 5 , and disability later in life 3 . The present findings (from a young, healthy cohort) thereby extend previous findings (from older, less healthy cohorts) suggesting that in absolute terms, individuals with the highest HGS have greater amounts of lean mass.

In addition to absolute HGS, we measured relative HGS by dividing absolute HGS by body weight (kg/kg), to normalize muscular strength and eliminate the overall size bias. A previous study performed on>1 million Swedish young men demonstrated that the handgrip/body weight ratio was a strong predictor of disability and musculoskeletal disorders later in life 10 . Similarly, relative HGS thresholds for diabetes risk in middle-aged adults (mean age ~33 years) was estimated at 0.78 for males and 0.57 for females using data extracted from the National Health and Nutrition Examination Survey (NHANES) 13 . Relative HGS cut-offs for metabolic syndrome (MetS), from data obtained from 1795 Columbian college students, demonstrated that 34.6% of the weakest males (relative HGS<0.466) had MetS, while 18.0% of weakest females (relative HGS<0.437) had MetS 14 . Absolute grip strength failed to show similar associations with MetS 22 .

Data on relative HGS from the current study extends these diabetes risk and MetS threshold data 13 14 by identifying a threshold for optimal health rather than disease. We found that a relative HGS>1.2 (1.3 for males and 1.1 for females, whereas an individual’s absolute grip strength exceeded body weight) was congruent with markers of good metabolic health ( Table 1 ) and physical fitness ( Table 2 ). Moreover, the strong negative association between relative HGS versus body fat percentage ( Fig. 2b ) highlights the probability, confirmed by linear modeling in the current study, that excess body fat reduces relative HGS (<1.0) towards previously estimated cut-off thresholds that predict both diabetes 13 and MetS 14 . Thus, our data concur with prior conclusions that increased body weight is the strongest predictor of disability later in life 10 but offers additional clarity by adding that body fat compartmental expansion likely serves as the primary mediator for both metabolic disease 13 14 23 and disability 10 .

Participants in the current study were largely young, metabolically healthy, and physically fit. The average BMI was within the normal range (<24.9 kg/m ^2^ ), with males slightly over the overweight threshold (25.3 kg/m ^2^ ) likely due to increased lean mass 24 . Metrics of metabolic health such as blood pressure, RHR, VAT, and BG levels were within the normal ranges 25 26 27 , with the exception of slightly elevated resting SBP values (129 mmHg) in our male cohort. Previous studies conducted in older (~65 years) populations document inverse relationships between HGS and hypertension 9 . Our data showed unexpected positive correlations between both absolute and relative HGS versus SBP, which contradicts many studies that suggest that resistance training decreases blood pressure 28 . Any potential negative effects of increased muscle strength on blood pressure in fit young males thereby requires further study.

In addition to our participants being metabolically healthy, the average VO _2_ max values for both the male (47 ml/kg/min) and female (40 ml/kg/min) cohorts would categorize their aerobic fitness between “fair” and “good” 17 . Similarly, the average vertical jump values for males (23”) and females (17”), would classify them as average or above-average in comparison with other young healthy adults 29 . Coupled with average absolute HGS values that were classified as “good” (males) and “excellent” (females), we confirm that our convenience sample was physically fit in addition to being metabolically healthy.

Lastly, significant, positive relationships (medium effect size, Table 3 ) between relative HGS verses both VO _2_ max and vertical jump (lower extremity power) suggests that normalized HGS may be a surrogate marker for overall physical fitness – not just localized forearm strength. This finding aligns with previous studies conducted on older adults (>65 years), whereas HGS estimated not only aerobic fitness 7 8 but also flexibility, balance and coordination, and overall physical fitness 7 . Therefore, we suggest that relative HGS be viewed as a more holistic marker of overall fatness and fitness than absolute HGS. Improvements in relative HGS would thereby require improvements in overall metabolic health (decrease body fat) and general physical fitness (aerobic fitness, upper and lower body strength improvements) to improve low relative HGS values (<1.0) into a healthier range (>1.0).

The limitations of the current study include a bias towards a physically fit sample. Our cross-sectional screening mainly attracted participants who were already physically active, despite our best efforts to recruit more sedentary college students. Our skewed sample of fit individuals, who exercise regularly, limits the broader interpretation of these findings to more sedentary populations. Nonetheless, our findings complement the large body of literature collected from older, diseased populations. One additional limitation was our assumption that the Handexer dynamometer was accurate (because it was FDA-approved). As such we did not perform any additional calibrations during data collection to ensure these data were accurate against a known standard.

In conclusion, our regression analyses and linear models confirm that higher absolute HGS is strongly biased towards larger, more muscular individuals. Conversely, higher relative HGS is most strongly biased towards individuals with lower body fat percentages. Our “average” data (means±SD), for all health and performance measures, would suggest that a relative HGS>1.0 (whereas an individual’s grip strength exceeds body weight) may be a quick, simple, cost-effective, and clinically useful indicator of overall metabolic health and physical fitness in young adults, worthy of further study as a holistic marker of health.

## References

[1] Soysal P Hurst C Demurtas J Handgrip strength and health outcomes: Umbrella review of systematic reviews with meta-analyses of observational studies J Sport Health Sci 2021 10 290 295 10.1016/j.jshs.2020.06.009 32565244 PMC8167328

[2] López-Bueno R Andersen L L Calatayud J Associations of handgrip strength with all-cause and cancer mortality in older adults: a prospective cohort study in 28 countries Age Ageing 2022 51 afac117 10.1093/ageing/afac117 35639798 PMC9351371

[3] Henriksson H Henriksson P Tynelius P Muscular weakness in adolescence is associated with disability 30 years later: a population-based cohort study of 1.2 million men Br J Sports Med 2019 53 1221 1230 10.1136/bjsports-2017-098723 29921654

[4] Esteban-Cornejo I Ho F K Petermann-Rocha F Handgrip strength and all-cause dementia incidence and mortality: findings from the UK Biobank prospective cohort study J Cachexia Sarcopenia Muscle 2022 13 1514 1525 10.1002/jcsm.12857 35445560 PMC9178163

[5] Celis-Morales C A Welsh P Lyall D M Associations of grip strength with cardiovascular, respiratory, and cancer outcomes and all cause mortality: prospective cohort study of half a million UK Biobank participants Bmj 2018 361 k1651 10.1136/bmj.k1651 29739772 PMC5939721

[6] López-Bueno R Andersen L L Koyanagi A Thresholds of handgrip strength for all-cause, cancer, and cardiovascular mortality: A systematic review with dose-response meta-analysis Ageing Res Rev 2022 82 101778 10.1016/j.arr.2022.101778 36332759

[7] Kim S H Kim T Park J C Usefulness of hand grip strength to estimate other physical fitness parameters in older adults Sci Rep 2022 12 17496 10.1038/s41598-022-22477-6 36261687 PMC9581452

[8] Sugie MH K Takahashi T Nara M Relationship between hand grip strength and peak VO2 in community-dwelling elderly outpatients JCSM Clinical Reports 2018 3 1 10 10.17987/jcsm-cr.v3i1.48

[9] Polo-López A Calatayud J Núñez-Cortés R Dose-response association between handgrip strength and hypertension: A longitudinal study of 76,503 European older adults Curr Probl Cardiol 2023 48 101813 10.1016/j.cpcardiol.2023.101813 37209803

[10] Ropponen A Silventoinen K Tynelius P Association between hand grip/body weight ratio and disability pension due to musculoskeletal disorders: a population-based cohort study of 1 million Swedish men Scand J Public Health 2011 39 830 838 10.1177/1403494811424610 21969330

[11] Yi D W Khang A R Lee H W Relative handgrip strength as a marker of metabolic syndrome: the Korea National Health and Nutrition Examination Survey (KNHANES) VI (2014-2015) Diabetes Metab Syndr Obes 2018 11 227 240 10.2147/dmso.S166875 29872330 PMC5973429

[12] Silva C R Saraiva B Nascimento DD C Relative handgrip strength as a simple tool to evaluate impaired heart rate recovery and a low chronotropic index in obese older women Int J Exerc Sci 2018 11 844 855 29997730 10.70252/WYJH9813 PMC6033494

[13] Brown E C Buchan D S Madi S A Grip strength cut points for diabetes risk among apparently healthy U.S. adults Am J Prev Med 2020 58 757 765 10.1016/j.amepre.2020.01.016 32273132

[14] Garcia-Hermoso A Tordecilla-Sanders A Correa-Bautista J E Muscle strength cut-offs for the detection of metabolic syndrome in a nonrepresentative sample of collegiate students from Colombia J Sport Health Sci 2020 9 283 290 10.1016/j.jshs.2018.09.004 32444153 PMC7242216

[15] Cronin J Lawton T Harris N A brief review of handgrip strength and sport performance J Strength Cond Res 2017 31 3187 3217 10.1519/jsc.0000000000002149 28820854

[16] Centers for Disease Control (CDC) and Prevention. 2021 Body Composition Procedures Manual. 2021 May: 1–120

[17] American College of Sports Medicine ACSM's Health-Related Physical Fitness Assessment (American College of Sports Medicine) 5th Edition Philadelphia PA Wolters Kluwer Medicine 2018

[18] Scrimgeour A G Noakes T D Adams B The influence of weekly training distance on fractional utilization of maximum aerobic capacity in marathon and ultramarathon runners Eur J Appl Physiol Occup Physiol 1986 55 202 209 3699009 10.1007/BF00715006

[19] Johnson S L Stone W J Bunn J A New author guidelines in statistical reporting: Embracing an era beyond p < .05 Int J Exerc Sci 2020 13 1 5 33042362 10.70252/HMZN3851 PMC7523905

[20] Sullivan G M Feinn R Using effect size--or why the p value is not enough J Grad Med Educ 2012 4 279 282 10.4300/jgme-d-12-00156.1 23997866 PMC3444174

[21] Lee S H Gong H S Measurement and interpretation of handgrip strength for research on sarcopenia and osteoporosis J Bone Metab 2020 27 85 96 10.11005/jbm.2020.27.2.85 32572369 PMC7297622

[22] Byeon J Y Lee M K Yu M S Lower relative handgrip strength is significantly associated with a higher prevalence of the metabolic syndrome in adults Metab Syndr Relat Disord 2019 17 280 288 10.1089/met.2018.0111 30945974

[23] Unamuno X Gómez-Ambrosi J Rodríguez A Adipokine dysregulation and adipose tissue inflammation in human obesity Eur J Clin Invest 2018 48 e12997 10.1111/eci.12997 29995306

[24] Weir C B Jan A BMI classification percentile and cut off points. In StatPearls Treasure Island (FL) StatPearls Publishing 2024 31082114

[25] Moebus S Göres L Lösch C Impact of time since last caloric intake on blood glucose levels Eur J Epidemiol 2011 26 719 728 10.1007/s10654-011-9608-z 21822717 PMC3186886

[26] American Diabetes Association 2. Classification and diagnosis of diabetes: Standards of medical care in diabetes-2018 Diabetes Care 2018 41 S13 S27 29222373 10.2337/dc18-S002

[27] American Heart Association. Understanding blood pressure readings. Available onlinehttps://www.heart.org/en/health-topics/high-blood-pressure/understanding-blood-pressure-readingsAccessed: 10-23-2024)

[28] Edwards J J Deenmamode AH P Griffiths M Exercise training and resting blood pressure: a large-scale pairwise and network meta-analysis of randomised controlled trials Br J Sports Med 2023 57 1317 1326 10.1136/bjsports-2022-106503 37491419

[29] Patterson D Peterson D F Vertical jump and leg power norms for young adults MSSE 2004 36 S114

